# MyVoice National Text Message Survey of Youth Aged 14 to 24 Years: Study Protocol

**DOI:** 10.2196/resprot.8502

**Published:** 2017-12-11

**Authors:** Melissa DeJonckheere, Lauren P Nichols, Michelle H Moniz, Kendrin R Sonneville, VG Vinod Vydiswaran, Xinyan Zhao, Timothy C Guetterman, Tammy Chang

**Affiliations:** ^1^ Department of Family Medicine University of Michigan Ann Arbor, MI United States; ^2^ Department of Obstetrics & Gynecology University of Michigan Ann Arbor, MI United States; ^3^ Institute for Healthcare Policy & Innovation University of Michigan Ann Arbor, MI United States; ^4^ Department of Nutritional Sciences School of Public Health University of Michigan Ann Arbor, MI United States; ^5^ Department of Learning Health Sciences University of Michigan Ann Arbor, MI United States; ^6^ School of Information University of Michigan Ann Arbor, MI United States

**Keywords:** adolescents, text messaging, longitudinal study, mixed methods, health policy

## Abstract

**Background:**

There has been little progress in adolescent health outcomes in recent decades. Researchers and youth-serving organizations struggle to accurately elicit youth voice and translate youth perspectives into health care policy.

**Objective:**

Our aim is to describe the protocol of the MyVoice Project, a longitudinal mixed methods study designed to engage youth, particularly those not typically included in research. Text messaging surveys are collected, analyzed, and disseminated in real time to leverage youth perspectives to impact policy.

**Methods:**

Youth aged 14 to 24 years are recruited to receive weekly text message surveys on a variety of policy and health topics. The research team, including academic researchers, methodologists, and youth, develop questions through an iterative writing and piloting process. Question topics are elicited from community organizations, researchers, and policy makers to inform salient policies. A youth-centered interactive platform has been developed that automatically sends confidential weekly surveys and incentives to participants. Parental consent is not required because the survey is of minimal risk to participants. Recruitment occurs online (eg, Facebook, Instagram, university health research website) and in person at community events. Weekly surveys collect both quantitative and qualitative data. Quantitative data are analyzed using descriptive statistics. Qualitative data are quickly analyzed using natural language processing and traditional qualitative methods. Mixed methods integration and analysis supports a more in-depth understanding of the research questions.

**Results:**

We are currently recruiting and enrolling participants through in-person and online strategies. Question development, weekly data collection, data analysis, and dissemination are in progress.

**Conclusions:**

MyVoice quickly ascertains the thoughts and opinions of youth in real time using a widespread, readily available technology—text messaging. Results are disseminated to researchers, policy makers, and youth-serving organizations through a variety of methods. Policy makers and organizations also share their priority areas with the research team to develop additional question sets to inform important policy decisions. Youth-serving organizations can use results to make decisions to promote youth well-being.

## Introduction

Youth are central to every major health problem today [1]. Addressing the health and well-being of youth can influence their attitudes, beliefs, and behaviors that ultimately impact health outcomes throughout their life span [2-8]. For example, behaviors that influence chronic disease such as diet and exercise are developed and reinforced during youth [9-11]. Further, symptoms of mental illness often manifest before age 25 [12,13], and sexual health issues are also prominent during adolescence. Despite the need to influence and improve youth health and behavior, there has been little progress in adolescent health outcomes in recent decades [1,14]. Initiatives to address poor health outcomes have largely been unsuccessful [15-18], likely because they are not informed by the adolescent perspective [1]. Instead, program and policy recommendations rely heavily on the perspectives of adults because youth often exert little autonomy in society and in health care settings [19-21].

Previous efforts to gather the perspectives of youth to investigate adolescent health have employed more traditional quantitative and qualitative data collection methods. For example, studies that have employed quantitative strategies, such as surveys, are challenged by a long turnaround time for collection, analysis, and dissemination [8,22,23]. In other studies, researchers have used qualitative focus groups and individual interviews to understand the contextual experiences of youth, or the how and why particular health problems and phenomena persist. However, sample sizes in qualitative studies are relatively small and therefore hard to scale or make inferences about the broader population that can inform policy [24,25]. Finally, many of these approaches rely on samples from secondary school, university, clinic, or community settings that underrepresent low-income, minority, and out-of-school individuals [3,4]. This suggests that adolescent health research has largely ignored the perspective of diverse groups of youth, limiting a full understanding of the issues facing adolescents and perpetuating health disparities [26,27]. In sum, the quality of data collected about this age group is lacking and translation into clinical practice or policy recommendations may be misguided. Research is needed that engages youth—particularly high-risk youth left out of traditional research—to understand their perspectives on health policies and practices that impact their lives to ultimately improve the health and well-being of youth.

One promising method for engaging youth in research is through text messaging. Studies that employ text messaging support the use of this novel strategy for data collection, concluding that the method is effective, easy to implement, and preferable among low-income communities [28,29]. This method seamlessly integrates into the day-to-day lives of youth, not adding the unnecessary burden of travel or time. In addition, text messaging has also been shown to result in more candid responses than voice interviews, even to sensitive questions [30]. As a result, text messaging offers an innovative, youth-friendly way to significantly increase access to and participation of low-income, high-risk, and otherwise excluded populations.

The use of text messaging can also address the problems with more traditional approaches (eg, interviews, surveys, school-based samples) to adolescent research described previously [28,31,32]. Text messaging is used by nearly all adolescents in the United States (97%) and is their preferred mode of communication due to its convenience and efficiency [33,34]. In contrast to quantitative survey methods, which have a long turnaround time and therefore can fail to influence critical or urgent policy decisions, text messaging has the potential to be used for real-time feedback on current health issues [35]. Unlike qualitative approaches such as interviews and focus groups, data collection via text messaging allows for rapid data collection and analysis for a large population-based sample allowing researchers to make generalizations that can inform policy and practice. Finally, rather than relying on school, clinical, or nonrepresentative community samples, text messaging is convenient and accessible to the vast majority of youth.

The MyVoice study was designed in response to calls for “strategic science,” defined as research that addresses gaps in knowledge important to policy decisions. Strategic science is research derived from the reciprocal flow of information between researchers and policy makers, and communicated not only in scholarly publications but also in forms relevant to policymakers [36].

MyVoice collects real-time data from youth via text messaging. The purpose of MyVoice is to harness youth perspectives to connect youth attitudes, beliefs, and behaviors to researchers and policymakers who can bridge the divide between youth and youth-centered policies. The aim of this paper is to describe the research protocol used to engage youth in an ongoing longitudinal research study to investigate youth perspectives on health and well-being. In addition, plans for dissemination of findings to inform policies that impact youth are described.

## Methods

### Overview

**Figure 1:**
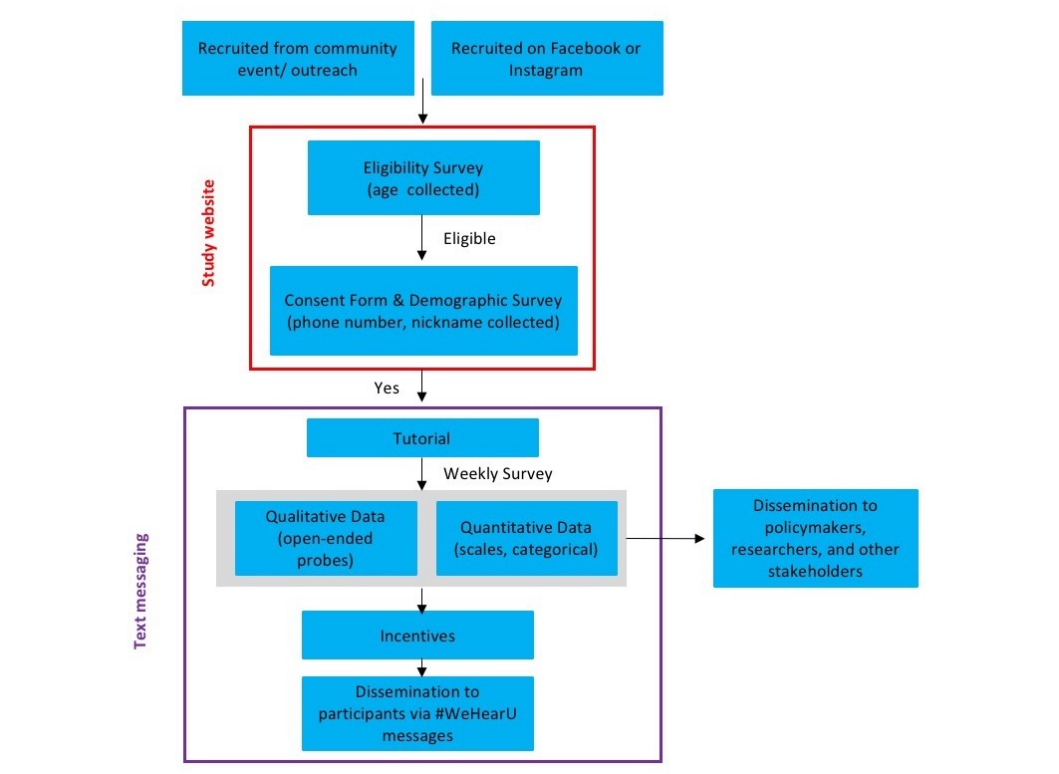
MyVoice research study design.

MyVoice uses a longitudinal mixed methods research design [37] to gain insight into youth perspectives on health, health care policy, and related issues important to the well-being of adolescents and young adults (see Figure 1). We seek to gain insight into matters that impact young people by gathering the voices of those directly affected by programs and policies—youth themselves. Each week, we will ask youth to respond to quantitative and qualitative survey questions via text message. The results will be quickly analyzed in real time and disseminated to youth, community members, academic researchers, and policymakers. In this way, the perspectives of youth can be central to the decisions made by community organizations, program directors, researchers, and lawmakers.

We assembled an interdisciplinary team of clinicians, academic investigators, methodologists, policymakers, and youth to bring together clinical, topical, methodological, and lived experiences relevant to youth health. The research team includes high school and college students to ensure that youth perspectives are incorporated into the full research process [38,39]. Youth research team members take on leadership roles, participate in decision-making processes, lead and support manuscript writing, generate research questions, support data collection and analysis, and create dissemination strategies. This study has been approved by the University of Michigan Institutional Review Board (HUM00119982).

### Project Development and Timeline

The MyVoice study has been iteratively developed through a yearlong pilot study from June 2016 to June 2017. The first phase of pilot testing was used to understand how to recruit and maintain a racially and economically diverse sample, and how to maximize the user experience for participants. The first phase was tested with 114 individuals and revealed key insights that were applied to improve the project. For example, we collected quantitative and qualitative evaluation data about recruitment and consenting procedures, participant preferences for style and content of questions, and technical glitches encountered while participating in the pilot phase. Although the full findings of the pilot evaluation are beyond the scope of this protocol, results were used to make the following changes to the study design: (1) focusing questions on political topics and current events, (2) waiving parental consent for participants younger than age 18 years, (3) addressing technical issues with the text messaging platform, and (4) using targeted advertisement to meet sampling quotas for demographics.

The next phase aims to enroll a national population-based sample of approximately 1000 participants. To date, MyVoice has enrolled approximately 800 participants and is nearing quota goals for all demographics.

### Recruitment

Youth are recruited in person, at community and youth-centered events, and online via Facebook and Instagram advertisements. Recruiting on Facebook and Instagram is an effective way to reach populations not traditionally reached through research because Facebook is used by 71% of adolescents and more likely to be used by teens that are from disadvantaged households [40]. Facebook and Instagram allows for targeted recruitment of specific populations, including low-income, minority youth, and youth not in high school or college. Eligibility criteria include age 14 to 24 years, ability to read and communicate in written English, and use of a phone with text messaging capabilities. Phones with text message capabilities from any operating system and carrier are able to participate. If eligible, consent is obtained from participants and parental consent is not required. Participants are only asked to report their nickname and their cell phone number, which is used only to send weekly surveys but are removed from the dataset before analysis to ensure confidentiality. At the time of enrollment, participants are sent a link to an online demographic survey (see Table 1). Demographic survey items include validated measures for gender, race, zip code, highest level of education, highest level of parent/guardian education, and an age-appropriate measure of socioeconomic status. Participants receive weekly surveys until they actively unenroll or when they reach age 25 years.

### Sampling

Quota sampling is used to achieve a population-based sample of youth participants. Recruited samples are matched with demographic characteristics of a national sample of youth aged 14 to 24 years. Facebook advertisements are created to target specific demographic characteristics, including age, gender, race/ethnicity, education, family income, and region of the country using weighted samples from the most recent American Community Survey. Creating a national sample of youth requires regular monitoring of participant demographics and tailoring online advertisements to meet predetermined quotas. Specifically, the study team is (1) opening recruitment on Facebook and Instagram and conducting weekly checks of participant demographics currently enrolled; (2) adjusting Facebook and Instagram recruitment advertisements to meet benchmarks, such as pausing advertisements targeting groups that have met the benchmark while increasing advertisements targeting populations that have not met the benchmark; and (3) continuing recruitment until all benchmarks are met and the sample is adequately powered for quantitative calculations.

### Incentives

Modest incentives are provided for participation throughout the study. Participants receive a one-time US $5 incentive for completing the online demographic survey on the study website following enrollment in the study. For the weekly surveys, participants receive US $1 for each completed survey. Every 12 weeks, participants also receive a US $3 bonus if all 12 weekly surveys are completed (total incentive of US $15 every 12 weeks for completed surveys). MyVoice also offers occasional “bonus” questions to elicit perspectives on time-sensitive policy issues. Participants receive an additional US $1 for completion of each bonus survey.

### Longitudinal Engagement

Based on our iterative pilot phases, we developed three primary strategies beyond the incentives described previously to promote participation and reduce attrition. First, the study design focuses on the perspectives of youth and asks youth to share their experiences. Second, with a goal to better understand and improve the health of youth, we actively generate weekly question sets that address topics that have real-world implications. Third, we regularly disseminate results to the youth participants, through infographics, summaries of findings, or select quotations (see Dissemination for more detail).

### Question Development

#### Generating

In weekly MyVoice research team meetings, upcoming topics are identified and discussed. A research question is identified to structure each week’s question set. Each week of questions focuses on one topic or issue identified by the MyVoice team, external youth-serving organizations, researchers, or policy makers. Topics are selected that align with upcoming policy priorities or timely policy concerns specific to the health of youth. In pilot testing, topics that have been fielded included stress, weight, nutrition, substance use, health insurance, relationships, and sexual health education.

#### Writing

Weekly text message surveys consist of three to five questions. Although topics and question structure varied, we include closed- and open-ended questions that assess the knowledge, attitudes, and beliefs of study participants. During team meetings, we revise questions to maximize comprehension, appropriateness for youth, and adherence to the research question. The team also discusses how to structure the questions to optimize the use of the text messaging platform.

#### Piloting

Once written and edited by the research team, weekly surveys are sent to a pilot team of youth, community members, survey experts, methodologists, and topical experts. Participants in the pilot-testing sample provide feedback regarding topics, sentence structure, phrasing, and word choice. The research team makes modifications to weekly surveys in response to pilot feedback before data collection.

### Data Collection

#### Tutorial

**Figure 2:**
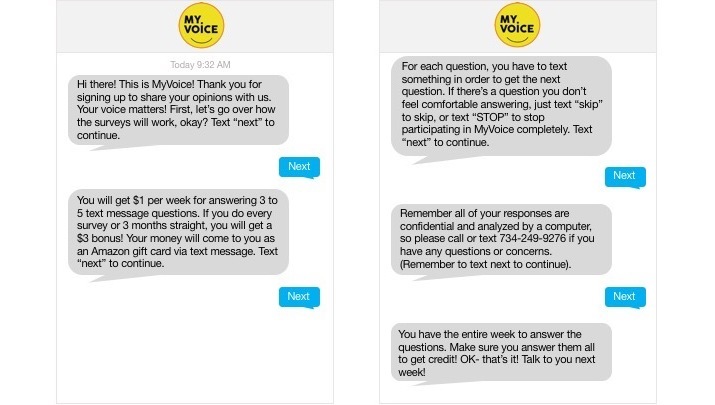
Screenshot of tutorial sent to new participants.

After participants provide consent/assent via the study website, they receive a text message tutorial that briefly describes the study process (see Figure 2). Participants are reminded that their participation is voluntary, responses are confidential, and any question can be skipped. In addition, the MyVoice tutorial provides a telephone number where study staff can be reached.

#### Weekly Survey

Questions are created in 12-week blocks. Textizen is the Web-based platform used to deliver surveys via text message. One-week delays are placed between each weekly question set. Weekly surveys are sent out to participants via Textizen on a consistent day and time.

#### Question Rating

Each week’s questions are rated by respondents on a scale of 1 to 5 “stars.” Ratings for questions are used to inform future surveys.

### Data Analysis

All responses are downloaded as a comma-separated values file. Data analysis depends on the nature of the questions from each weekly survey. Closed-ended and categorical (quantitative) responses are cleaned for case mismatch, spelling variants, and typographic errors to prepare for analysis using descriptive statistics.

**Table 1:** MyVoice demographic survey completed on enrollment.

Question		Response options	
What is your date of birth? Please enter it in the following format: mm/dd/yyyy.		Open-ended	
What is your cell phone number? Please enter in the following format: 1 555 555 5555		Open-ended	
What is your gender?		Male; female; transgender (FTM); transgender (MTF); nonbinary; other (please specify)	
What is your race? Check all that apply.		American Indian or Alaska Native; Asian; black or African American; Native Hawaiian or other Pacific Islander; white or Caucasian; other (please describe)	
Are you Hispanic or Latino?		Yes; no	
What zip code do you live in?		Open-ended	
What is the highest level of education you have achieved?		Eighth grade or less; some high school; high school graduate; some vocational/technical training; completed vocational/technical training; some college; completed an associate’s degree; completed a bachelor’s degree; some graduate school; completed a master’s degree; some graduate training beyond a master’s degree; completed a doctoral degree	
What is the highest level of education any parent/guardian has achieved?		Eighth grade or less; some high school; high school graduate; some vocational/technical training; completed vocational/technical training; some college; completed an associate’s degree; completed a bachelor’s degree; some graduate school; completed a master’s degree; some graduate training beyond a master’s degree; completed a doctoral degree	
Who do you live with most of the time?		My parent(s)/guardian(s); my aunt/uncle; my kids; in a dorm; in apartment or house with other people, not family; in a fraternity/sorority; I live alone; other; my spouse, partner, or significant other	
How many people are in your immediate family? (Include you, any parents/guardians, siblings, step-siblings, etc)		1-3; 4-6; 7-10; ≥10	
What is your parent(s)/guardians’ current marital status?		Married; together but not married; separated; divorced; widowed; unsure	
How did you hear about MyVoice?		From a family member; from a friend; Facebook; Instagram; other	
**Socioeconomic status measure for respondents under age 18 (version received determined by participant’s date of birth)**			
	Thinking about the house you live in at the moment, do your parents own it or rent it? (If they have a mortgage, tick “they own it”)		They own it; they rent it; I don’t know
	Do you have a car or van at home?		Yes, one car or van; yes, more than one car or van; no, we don’t own a car or van
	Which of the following Internet technology devices do you have at home?		Desktop computer; laptop computer; iPad or other tablet; other (please specify)
	When you were in middle/high school, did you receive free or reduced price school lunch?		Yes; no
**Socioeconomic status measure for respondents age 18 and older (version received determined by participant’s date of birth)**			
	What is your annual household income? (Just an estimate of the total amount of everyone in your household)		Open-ended

**Figure 3:**
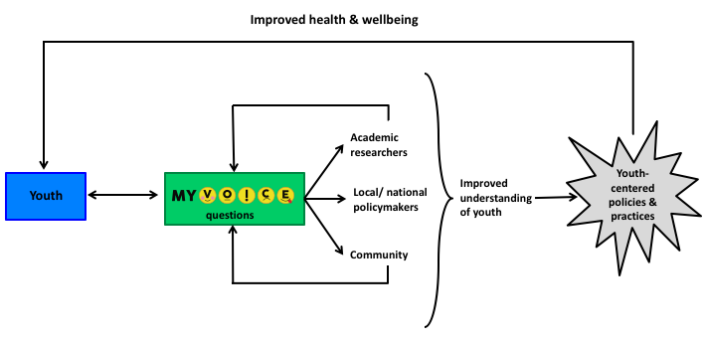
MyVoice dissemination model.

For open-ended (qualitative) responses, natural language processing (NLP) techniques are applied to cluster responses. Using NLP, responses are parsed and synonymous words are first grouped into word clusters. These word clusters are further grouped using word similarity measures resulting in semantic word clusters. Our initial analysis of these semantic word clusters shows sufficient agreement with traditional qualitative methods based on manual review and coding responses. Effectiveness of this approach will be reported in a future paper.

Results may be stratified by demographic characteristics to better understand differences and meet the needs of specific stakeholder organizations. For example, responses to surveys may be stratified by age to distinguish between younger (age 14-18 years) and older (age 19-24 years) respondents.

### Dissemination

Results will be disseminated to multiple audiences, including academics, policymakers, and youth (see Figure 3). Dissemination products include media/online graphics or GIFs, presentations to community groups, one-page reports, policy briefs, academic presentations, and academic manuscripts. These products are determined by the audience and specific study and content area being disseminated. For example, a lawmaker interested in findings related to beliefs about sexually transmitted diseases may request a one-page infographic to be distributed during community town halls to influence local policy, whereas a study on eating behavior may be reported in a peer-reviewed manuscript.

**Figure 4:**
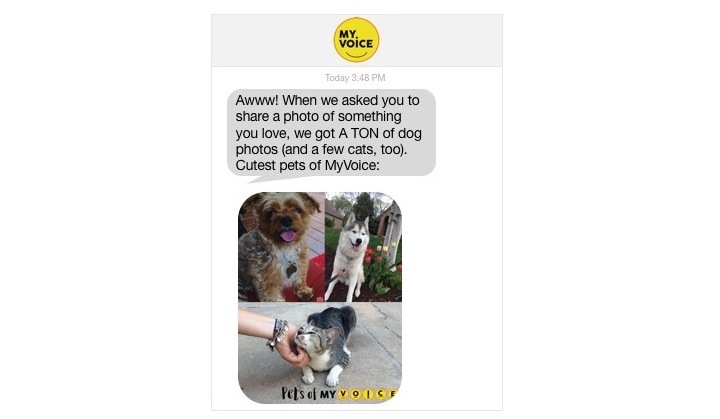
Example of #WeHearU sent to participants.

**Figure 5:**
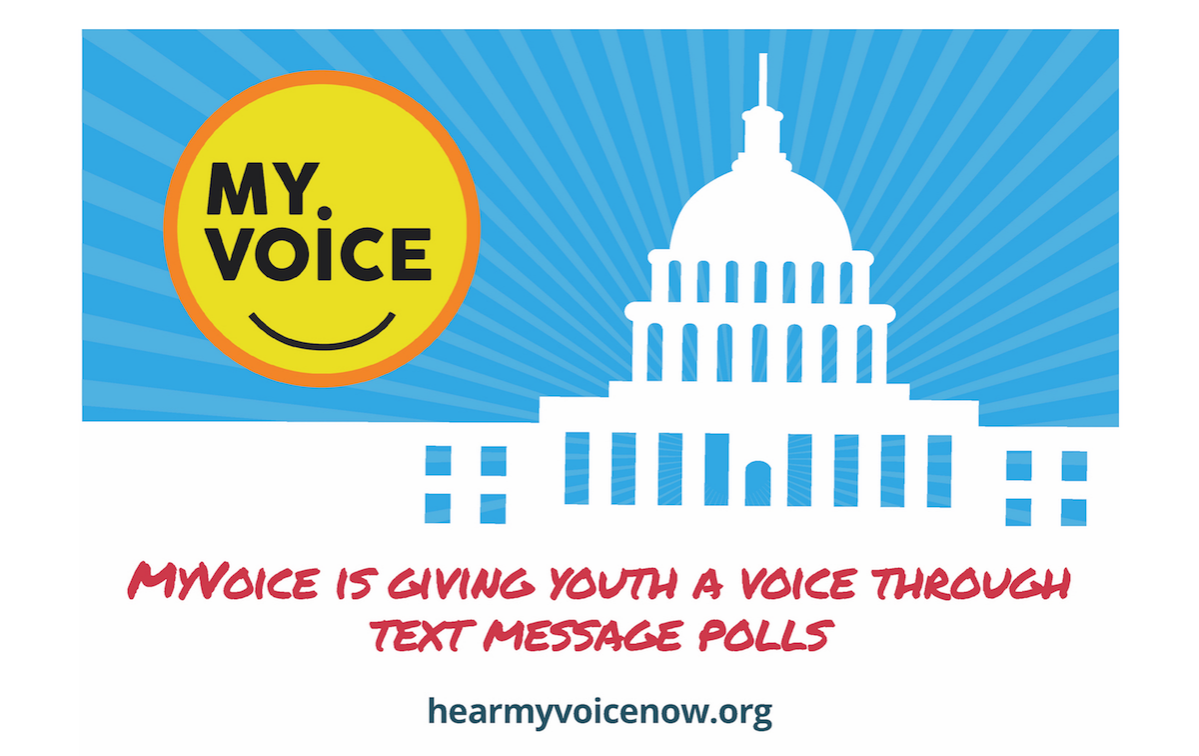
Postcard distributed to lawmakers.

For youth, findings are shared via text message as #WeHearU messages (see Figures 4 and 5). Following data analysis, #WeHearU infographics or narratives are created to build engagement, encourage participation, and share the depth and breadth of responses. We also demonstrate the commitment of the MyVoice team to translate the words of youth into messages for lawmakers and youth-serving organizations by creating and distributing postcards featuring summaries of results or quotes from study participants (see Figure 5).

## Results

**Figure 6:**
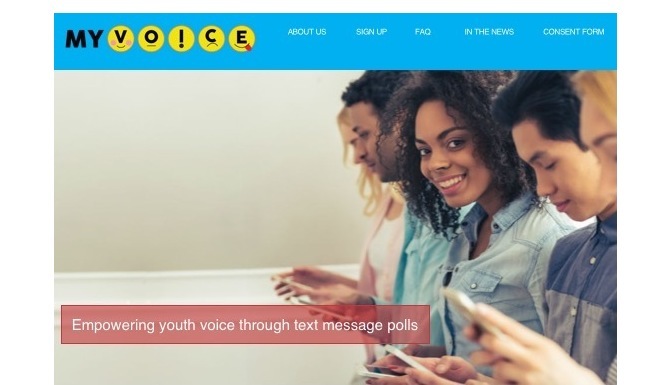
Screenshot of home page for MyVoice website.

**Figure 7:**
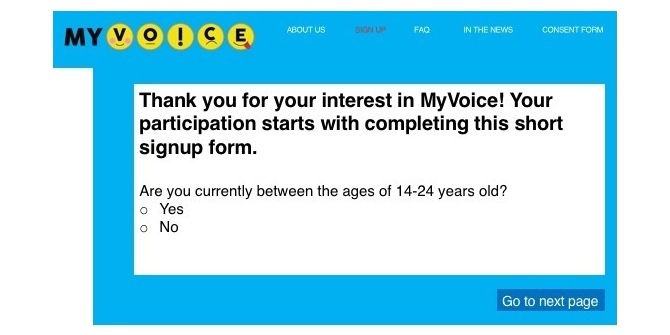
Embedded enrollment (including consent) form in MyVoice website.

We have launched the national study and are currently recruiting and enrolling participants through in-person and online strategies. The MyVoice website is live and enrollment (including consent) and demographic questionnaires are embedded (see Figures 6 and 7). Question development, weekly data collection, data analysis, and dissemination are in progress.

Through the first two phases of pilot testing, we have collected 56 weeks of data from 114 participants. See Textbox 1 for a selection of topics and corresponding question sets sent to participants.

**Examples of MyVoice topics and questions.**

**Police**

Intro: *Hi, it’s MyVoice! Some people feel safer when police officers are around. Some people feel less safe. How do you feel when police officers are around you?*

Q1: *There has been news of unarmed people being shot by police. On a scale of 1 to 5 how big of a problem is this? (1=not a big prob; 5=a very big prob)*

Q2: *Why?*

Q3: *What are some things that police could do better to keep people safe?*

Q4: *What have your experiences with police been like?*

Q5: *Rate this week’s questions (1 through 5)! 1=one star, 5=five stars*

Outro: *That’s all for this week. Thank you!*

**Sexually transmitted diseases**

Intro: *Hi, {{name}}. We are interested in your thoughts about sexually transmitted infections (STIs). If you had an STI, would you tell your sexual partner(s)?*

Q1: *Why or why not?*

Q2: *Would it be hard for you to get TESTED for an STI? Why or why not?*

Q3: *Would it be hard for you to get TREATED for an STI? Why or why not?*

Q4: *Rate this week’s questions (1 through 5)! 1=one star, 5=five stars*

Outro: *You’re done for the week, {{name}}! Talk to you next week.*

**Student debt**

Intro: *Hi {{name}}! This week’s questions ask your thoughts about college. Whether you are in school or not, we want to hear your opinion. Type “next” to continue.*

Q1: *What are three things that might make it hard for you to go to college?*

Q2: *How much student loan debt is ok to have for a college education? Why?*

Q3: *Should college be free? Why or why not?*

Q4: *How much do you expect to earn per year in your first full-time job?*

Q5: *Rate this week’s questions (1-5)! 1=one star, 5=five stars*

Outro: *Alright, you are finished and made another dollar! We’ll talk to you next week! :)*

### Limitations

The MyVoice sample is not nationally representative; however, quota sampling allows MyVoice to recruit using a variety of methods while meeting national benchmarks for important demographic characteristics.

Question sets typically only include three to five questions per week and may require repeat longitudinal sampling to get in-depth knowledge (ie, fielding several weeks of questions on the same topic). However mixed methods techniques that integrate qualitative and quantitative data allow us to collect data with a deep understanding of context not attainable in traditional surveys of youth and are collected in a manner that may elicit more truthful responses [30].

## Discussion

This study represents a novel example of strategic science that collects youth thoughts and opinions in real time with a goal of disseminating to appropriate change agents at the pace that policy decisions are being made [36].

This study protocol describes a youth-centered method of gathering the beliefs, attitudes, and behaviors of youth in their own words. We know that sustainability of interventions, programs, and policies is dependent on the buy-in of its users. Understanding youth perspectives can allow for the development of youth-centered policies that build on the needs, priorities, and recommendations of those who will be directly affected.

The Department of Health and Human Services Office of Adolescent Health currently lists no ongoing longitudinal qualitative or mixed methods study of youth [41]. As a result, our study is expected to make several methodological contributions. First, we will engage a national population-based sample of youth in a longitudinal mixed methods study of youth that includes voices that are traditionally excluded from research (ie, populations) and policy recommendations.

Second, we have developed a strategy to engage youth over time. Including youth in longitudinal research has been difficult, yet essential, to understanding the health problems we face today [1]. We employ text messaging as an inclusive, comfortable, low-burden mechanism for participation [42,43].

Third, our use of text messaging and NLP allows for real-time data collection, analysis, and dissemination of research findings. The timeline of research (months to years) is typically misaligned with the timeline of policy decision making (weeks to months) [44]. MyVoice bridges this gap by creating a method that better matches the needs of policy makers at all levels with the goal of informing youth-centered policies at all levels.

Finally, our project adds to the growing evidence that participation of advisory groups, peer leaders, and community researchers enhances the quality and reach of research [45-47]. Youth play an active role on our research team. They have an ongoing role in the development of the study, recruitment of participants, data collection and analysis, and dissemination to diverse audiences. Alongside academic researchers, community organizations, and clinicians, youth are encouraged to participate in decision-making processes and take on leadership roles.

## Abbreviations

NLP: natural language processing
